# Epigenetic Alterations in Bladder Cancer and Their Potential Clinical Implications

**DOI:** 10.1155/2012/546917

**Published:** 2012-06-21

**Authors:** Han Han, Erika M. Wolff, Gangning Liang

**Affiliations:** ^1^Department of Pharmacology and Pharmaceutical Sciences, School of Pharmacy, University of Southern California, Los Angeles, CA 90033, USA; ^2^Department of Urology, Norris Comprehensive Cancer Center, Keck School of Medicine, University of Southern California, Los Angeles, CA 90033, USA

## Abstract

Urothelial carcinoma (UC), the most common type of bladder cancer, is one of the most expensive malignancies to treat due to its high rate of recurrence. The characterization of the genetic alterations associated with UC has revealed the presence of two mutually exclusive molecular pathways along which distinct genetic abnormalities contribute to the formation of invasive and noninvasive tumors. Here, we focus on the epigenetic alterations found in UC, including the presence of an epigenetic field defect throughout bladders with tumors. A distinct hypomethylation pattern was found in noninvasive tumors, whereas widespread hypermethylation was found in invasive tumors, indicating the two pathways given rise to two tumor types also differ epigenetically. Since certain epigenetic alterations precede histopathological changes, they can serve as excellent markers for the development of diagnostic, prognostic, and surveillance tools. In addition, their dynamic nature and reversibility with pharmacological interventions open new and exciting avenues for therapies. The epigenetic abnormalities associated with UC would make it an excellent target for epigenetic therapy, which is currently approved for the treatment of a few hematological malignancies. Future research is needed to address efficacy and potential toxicity issues before it can be implemented as a therapeutic strategy for solid tumors.

## 1. Introduction 

**Figure 1:**
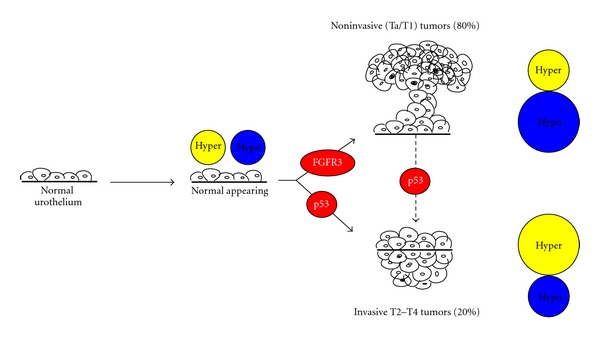
Two distinct molecular pathways for the initiation and progression of urothelial carcinoma. Normal urothelium acquire both aberrant DNA hypermethylation and hypomethylation, prior to the onset of genetic mutations. Normal-appearing urothelium then can transform into either noninvasive (Ta/T1) tumors or invasive tumors (T2–T4) through the accumulation of activating mutations of FGFR3 (fibroblast growth factor receptor 3) or *TP53*, respectively. Approximately, 80% of all newly diagnosed cases are noninvasive papillary tumors, which do not often progress to invasive tumors. Acquiring subsequently *TP53* mutation is necessary for the progression. Noninvasive tumors acquire less hypermethylation and more aberrant hypomethylation, among which a group of genes is distinctively hypomethylated in noninvasive tumors. Invasive tumors display the reversed methylation profile.

Bladder cancer is one of the most commonly diagnosed malignancies in the United States, with an estimated number of 73,510 new cases and 14,880 deaths in 2012 [1]. Worldwide, bladder cancer is the seventh most common malignancy [2]. The risk factors associated with development of bladder cancer include cigarette-smoking, exposure to chemicals, such as aromatic amines, chronic bladder inflammation, genetic predisposition, and age [3, 4]. In the United States, more than 90% of bladder tumors are diagnosed as urothelial carcinoma (UC), 5% as squamous-cell carcinoma (SCC), and 2% as adenocarcinomas [5]. In countries, where chronic urinary infection by *Schistosoma haematobium* is prevalent, most bladder cancers are SCC [6]. Due to the low incidence of SCC in the US as well as the rest of the Western countries, this paper primarily focuses on UC. Of all newly diagnosed UC cases, approximately 80% are noninvasive papillary tumors, which are confined to the urothelium (CIS, Ta) or lamina propria (T1). The remaining 20% of tumors are muscle invasive (T2–T4) and are typically treated by radical cystectomy [7]. Despite the fact that most noninvasive UCs can be successfully treated by transurethral resection of bladder tumor (TURBT), 70% of patients will suffer tumor recurrence after the initial treatment and 10–20% of those recurrent tumors will become invasive. Specific genetic alterations characterize UCs; for instance, noninvasive tumors show frequent mutations in fibroblast growth factor receptor 3 (FGFR3) mutations; whereas invasive tumors often display *TP53* mutations. Further progression of noninvasive tumors to invasive tumors requires subsequent mutations in *TP53* (Figure 1) [4, 8]. The high rate of recurrence and inability to predict which tumor will progress require frequent and invasive clinical management after the initial treatment.

Currently, the gold standard for bladder cancer diagnosis and surveillance is cystoscopy, which is an invasive and expensive method that allows direct visualization of the bladder. Noninvasive methods are also available, but the majority of them lack sensitivity. Urinary cytology is the most widely used noninvasive method for detecting the presence of cancerous cells in urine and is often used in conjunction with cystoscopy. However, this method shows poor performance in detecting low-grade tumors [5, 9]. Furthermore, the accuracy of urinary cytology is jeopardized by interobserver variability [5]. The current recommended post-TURBT surveillance regimen for tumor recurrence involves a combination of cystoscopy and voided urine cytology every three months for two years and once a year thereafter [10]. This results in $2.2 billion annual expenditure, making bladder cancer one of the most expensive malignancies to treat [4, 11, 12]. In recent years, much effort has been dedicated to the discovery of tumor biomarkers that represent tumor properties to overcome the limitations of cystoscopy and cytology. Although some progress has been made in this area with some biomarkers showing considerable clinical values, the majority of them lack sensitivity and/or specificity [13]. To date, no biomarker assay stands alone to detect and monitor the disease. Therefore, the elucidation of the molecular mechanisms that underlie the high rate of recurrence shown by bladder tumors will help to develop more accurate and cost-effective noninvasive strategies for diagnosis, prognosis, and surveillance of the disease.

## 2. Genetic Mutations Associated with Invasive and Noninvasive Urothelial Carcinoma 

Many types of invasive carcinomas, including colon cancer [14], arise from noninvasive carcinomas via the accumulation of mutations over time. However, pioneering work done by our group has demonstrated that such a developmental continuum does not exist in UC. There is substantial evidence for the existence of two mutually exclusive molecular pathways that lead to bladder carcinogenesis in which distinct genetic alterations are responsible for the formation of noninvasive and invasive tumors, resulting in divergent clinical behaviors [15]. Noninvasive tumors usually arise by tissue hyperplasia and show mutations in fibroblast growth factor receptor 3 (FGFR3) [16, 17], which is involved in cell differentiation and angiogenesis [18]. Patients with such tumors usually do not show disease progression, but experience frequent recurrence [8, 19]. Invasive tumors are believed to arise by tissue dysplasia and often harbor mutations in *TP53* [15, 20], a critical tumor suppressor gene that initiates cell-cycle arrest upon DNA damage [21]. These tumors are aggressive and associated with high mortality [8]. These two pathways do not occur sequentially and only under rare circumstances, when a subsequent p53 mutation is acquired, noninvasive tumors can progress to invasive tumors [15]. The genetic alterations associated with UC are relatively well defined as compared to its epigenetic alterations. Therefore, this paper mainly focuses on the epigenetic aberrations found in UC. 

## 3. The Epigenetic Landscape and Its ****Deregulation in Urothelial Carcinomas

Epigenetics encompasses the heritable changes in gene expression that are not caused by changes in the underlying DNA sequence [22]. Such epigenetic changes include DNA methylation, histone modifications, and nucleosome positioning [15, 22–24]. Among the three layers of epigenetic regulation, DNA methylation was the first to be identified and is the most extensively studied. It involves the covalent addition of a methyl group to the 5′ position of cytosine residues in the context of CpG dinucleotides. The distribution of CpG sites is asymmetrical and nonrandom throughout the genome, with a high frequency of CpG sites occurring near promoters (CpG islands) and repetitive elements [25, 26]. The majority of promoter-associated CpG islands are usually not methylated under normal conditions, with the exception of imprinted genes [25, 27]. DNA methylation at gene promoters modifies DNA accessibility to transcription factors or helps recruit silencing-associated proteins, resulting in gene silencing [28, 29].

The N-termini of histones undergo a variety of posttranslational modifications, including methylation, acetylation, phosphorylation, ubiquitination, and sumoylation to generate transcriptionally permissive or refractory chromatin conformations depending on the type and location of the modification [23, 30]. For instance, trimethylation of lysine 4 on Histone 3 (H3K4me3) is enriched at the promoters of transcriptionally active genes [31], whereas trimethylation of H3K9 and H3K27 is associated with transcriptionally inactive gene promoters [23]. The balanced activity of histone modifying enzymes that add or remove specific modifications is critical for normal cell physiology [32]. In addition, the presence of specific histone variants at regulatory regions also plays a role in controlling gene expression by influencing the stability of nucleosome occupancy [33], which either facilitates or prevents binding of transcription machinery at transcription start sites [34, 35]. 

In addition to genetic abnormalities, epigenetic alterations also play vital roles in the initiation as well as progression of cancer. Global disruption of the epigenetic landscape, resulting in aberrant gene expression and function, is a hallmark of human cancer [27, 36]. The cancer methylome is highly disrupted, featuring hypermethylation and aberrant silencing of tumor suppressor genes, and hypomethylation of repetitive sequences, transposons, and oncogenes, which contributes to tumorigenesis by increasing chromosomal instability and activating aberrant transcripts [36–38]. Substantial evidence shows that the epigenome of UC cells displays profound alterations in DNA methylation, histone modifications, and nucleosome positioning. In this context, a few well-known tumor suppressor genes, including *CDH1*, *CDH13*, *INK4A*, *RASSF1A*, *APC*, *ARF*, *MLH1*, and *DAPK* [39–41], have been reported to be frequently hypermethylated and silenced in UC, resulting in deregulated cell proliferation [42]. In addition to global hypomethylation of repetitive elements, such as long interspersed nuclear elements (LINE-1) [43], work done by our group has demonstrated that a specific LINE-1 located within the mesenchymal-epithelial transition factor (MET) oncogene (L1-MET) is hypomethylated and transcriptionally active in UC, accompanied by the presence of a nucleosome-depleted region (NDR) just upstream of the transcription start site (TSS), active histone marks, and the histone variant H2A.Z [44]. Recent advances in high-throughput technologies have facilitated the identification of distinct DNA methylation, gene expression, and histone modification profiles associated with tumors, including UC [45–48]. Such technologies will aid in establishing a comprehensive understanding of the altered epigenome present in the diseased state, and subsequently facilitate the identification of potential drug targets and biomarkers for diagnostic and prognostic purposes.

The two mutually exclusive molecular pathways for the formation of noninvasive and invasive tumors also differ epigenetically in addition to genetically. A genome-wide analysis of DNA methylation patterns in noninvasive and invasive urothelial tumors revealed a distinct hypomethylation pattern only in noninvasive tumors and widespread hypermethylation in invasive tumors, suggesting that they arise via distinct epigenetic pathways [46]. When correlations between DNA methylation and gene expression were performed, an inverse relationship was observed for most genes, highlighting the functional significance of both aberrant DNA hypermethylation and hypomethylation of gene promoters in tumors. Many of the hypomethylated loci distinctively associated with noninvasive tumors are non-CpG island promoters of tissue-specific genes. The unique hypomethylation pattern present in the noninvasive tumors may explain the failure of these tumors to become invasive [46].

## 4. Epigenetic Field Defect

 The alarmingly high recurrence rate of bladder cancer is of clinical concern, highlighting the need for physicians and scientists to elucidate its underlying mechanism. The presence of a field defect, an area of tissue that is predisposed to undergo oncogenic transformation, has been postulated to be responsible for such high recurrence rate [49]. This concept was first introduced by Slaughter et al.,who found abnormal tissues composed of epithelial cells of polyclonal origins surrounding oral squamous cell carcinomas [50]. Since then a field defect, as identified by genetic alterations, has been found in tumors arising from various tissues, including upper aerodigestive tract [51], lung [52], esophagus [53], vulva [54], cervix [55], colon [56], skin [57], and bladder [58, 59].

In addition to genetic field defects, epigenetic field defects have also been found in various types of cancer, including stomach [60, 61], liver [62], colon [63–65], lung [66], breast [67], kidney [68], and esophageal [69]. Using the Illumina GoldenGate assay to compare primary tumors, normal-appearing tissues at 0.5 cm increments away from the tumor in multiple directions, and urothelium from cancer-free bladders, our group found that cancer-bearing bladders have a widespread epigenetic field defect [46]. Methylation at a significant number of loci (169 probes spanning 155 unique gene regions) was altered not only in tumors but also in normal-appearing urothelial taken at least 5 cm away from the corresponding primary tumor, with the majority of the loci, such as *ZO2*, *MYOD1*, and *CDH13,* being aberrantly hypermethylated [46]. Among the 169 loci, 145 loci displayed a trend of increasing methylation in invasive tumors and 41 loci in noninvasive tumors, indicating that hypermethylation may constitute the majority of epigenetic defects present in the urothelium. In addition, we also observed hypomethylation and ectopic expression of L1-MET in primary tumors and surrounding histologically normal tissues [44]. Together, these studies suggest that uniquely hypermethylated or hypomethylated loci that are found in bladder tumors and surrounding tissues may serve as biomarkers and could be used to develop diagnostic, prognostic, and/or surveillance tools.

The field defect found in tumor-bearing bladders could be propagated by clonal expansion or a generalized epigenetic field defect. Clonal expansion involves the process of accumulating aberrant DNA methylation in one cell, followed by expansion of that cell population across the urothelium, resulting in subsequent transformation. Analysis of the pattern of X-chromosome inactivation, which is maintained during clonal expansion, in samples taken from 2 female patients indicated that the widespread epigenetic field defect observed in UC could not be attributed to clonal expansion. Instead, it is likely that epigenetic alterations occur independently in many cells across the urothelium, thereby predisposing them to undergo oncogenic transformation [46]. The urothelium is uniformly exposed to carcinogens, causing epigenetic alterations, initially without associated histological changes. It is plausible that at the initiation of UC, there is no “normal” urothelium present and this may provide an explanation for its high recurrence rate after TURBT. The altered epigenome in the normal-appearing urothelium may allow for a more permissive environment for the growth of newly transformed cells.

## 5. Using DNA Methylation as a Marker for ****Diagnosis, Prognosis, and Surveillance

 Since bladder cancer may remain asymptomatic until a relatively late stage, ideal clinical management would be comprised of early detection, accurate prediction of disease progression, and frequent monitoring. However, unlike many other types of cancers, there is no standard and effective noninvasive strategy for early detection [70]. Currently, conventional histopathological evaluations that are used for the categorization of tumor grade and stage are also used to predict the potential behavior of tumors. Such histopathological evaluations are not accurate in predicting the behaviors of heterogeneous tumors, resulting in significant differences in clinical outcomes for patients with tumors of similar stages [71]. Therefore, patients undergo frequent and long-term surveillance after the initial treatment. There is a strong need to develop economically viable, noninvasive methods with high sensitivity and specificity for diagnosis, prognosis, and monitoring of UC. A better understanding from both a genetic and an epigenetic perspective of how UC arises and progresses has greatly contributed to the ongoing efforts to create these new assays. 

 The ability to detect cancer-specific genetic and epigenetic alterations in cells detached from the urothelium, which can be found in voided urine samples, supports the use of such biomarkers in the development of noninvasive methods for bladder cancer detection and progression [40, 72–75]. Several of the most promising genetic biomarkers whose protein or expression levels are upregulated in the diseased state, including nuclear matrix protein 22 (NMP-22), telomerase, and the nuclear matrix protein bladder cancer 4 (BLCA-4), have been reported to have promising values [73]. However, they suffer from similar limitations as urine cytology—low sensitivity for low grade tumors. Although some of the markers have been used to complement cystoscopy and urinary cytology, none of them has been utilized independently [73]. The detection of genetic mutations DNA extracted from urine sediment is another screening method, and mutations of the fibroblast growth factor 3 (FGFR3) gene, which frequently occur in superficial bladder tumors, can be readily identified by this method, providing greater sensitivity in the detection of TA tumors than cytology [76]. 

A greater understanding of the roles epigenetics plays in tumorigenesis has opened up new avenues for developing innovative diagnostic and prognostic biomarkers. Because of their early onset in bladder tumorigenesis and presence in precancerous lesions and tissues surrounding primary tumors (field defect), DNA methylation changes are excellent biomarker candidates [46, 74]. Tumor-associated alterations in DNA methylation are readily detectable in body fluids, such as blood [77] and urine [72, 78]. We have shown that DNA isolated from urine and primary tumors of bladder cancer patients show similar methylation profiles, displaying hypermethylation at a number of apoptosis-associated genes, including *DAPK*, *BCL2,* and *TERT*. These loci are not methylated in urine specimens from healthy controls [72], suggesting that such tumor-specific methylation markers have the potential to serve as diagnostic tools using a noninvasive sample procurement method. Numerous studies have identified a number of additional methylation marks suited for urine-based detection, including the combination of TWIST and NID2 [79] and the combination of E-cadherin, p14, and RASSF1A [80]. Costa and collaborators reported 100% sensitivity and 94% of specificity for early stage Ta and low-grade UC when evaluating DNA methylation changes in a panel of 3 genes: *GDF15*, *TMEFF2,* and *VIM* [74]. Reinert and collaborators established a detailed mapping of the methylome in bladder cancer and identified four novel DNA methylation marks: *HOXA9*, *ZNF154*, *POU4F2,* and *EOMES *[75]. It is of interest that the methylation status of genes that show nontumor specific DNA methylation patterns can be potentially used for assessing prognosis and risk for recurrence. This category includes genes that are aberrantly methylated in histologically normal tissues surrounding bladder primary tumors, such as *ZO2*, *MYOD1,*and *CDH13* [46].

The technological advances in the detection of global methylation patterns have facilitated the characterization of tumor methylomes, thereby providing new opportunities to find better and more sensitive biomarkers. Although efforts in this regard are currently underway, more studies are needed to translate these findings into the clinical setting. 

## 6. Urothelial Carcinoma and Epigenetic ****Therapies 

Although epigenetic modifications are heritable, their dynamic nature and reversibility through pharmacological interventions make them excellent targets for anticancer therapies. Over the past few decades, various drugs aimed at targeting different types of epigenetic alterations observed in cancer, including DNA methylation and histone modifications, have been developed, with the goal of reactivating aberrantly silenced genes. In addition to having genetic abnormalities, UC is also driven by progressive alterations in the epigenome, resulting in changes in chromatin packaging and aberrant gene expression [46]. Epigenetic changes in UC have been well elucidated and their significance has been demonstrated, making UC a suitable candidate for epigenetic therapy. Due to the presence of an epigenetic field defect in UC, epigenetic therapies may also prevent recurrence by reversing the epigenetic aberrations occurring in histological normal tissues that remain after TURBT.

UC is an excellent candidate for epigenetic therapy due to the presence of a highly disturbed epigenome, which can be restored via the intervention of epigenetic agents. Promoter hypermethylation accompanied by histone modifications which facilitate the formation of heterochromatin is commonly seen in UC. DNA methyltransferase inhibitors (DNMTi) and/or histone deacetylase inhibitors (HDACi) could be used to reverse such abnormalities and restore the expression of aberrantly silenced genes. In addition to having therapeutic value, epigenetic therapies also have preventive value in patients who had undergone TURBT, which leaves large areas of epigenetically altered tissues. Our lab has demonstrated that *ZO2*, which is methylated in tumors and adjacent normal-appearing tissues, is reactivated upon 5-Aza-2-deoxcytidine (5-Aza-CdR) treatment in a panel of bladder cancer cell lines [46]. Treatment with DNMTi also has the potential to reverse the invasiveness of high-grade tumors by creating an epigenetic profile similar to that of low-grade tumors. As discussed above, noninvasive tumors show a unique hypomethylation pattern in the vicinity of TSSs which may account for their failure to acquire an invasive phenotype.

## 7. DNA Methyltransferase Inhibitors 

The widespread hypermethylation at promoters in UC, particularly in invasive tumors [40, 41, 46] suggests that restoration of a normal epigenome through the use of DNA hypomethylating agents would be clinically beneficial. Many of these agents are nucleoside analogues, which get incorporated into DNA and sequester DNA methyltransferases (DNMTs), resulting in depletion of DNMTs and global hypomethylation upon subsequent cell divisions [81]. 

Two DNA methylation inhibitors, 5-Azacytidine (5-Aza-CR; Vidaza) and 5-Aza-2-deoxycytidine (5-Aza-CdR; Decitabine), have been approved by the Food and Drug Administration (FDA) for the treatment of myeloid malignancies [81]. Both are cytosine analogues that are incorporated into replicating DNA in the place of cytosine, resulting in heritable global demethylation [32, 82]. In addition, 5-Aza-CR is also incorporated into RNA, which prevents the translation of oncogenic proteins [83, 84].

Despite their promising results in treating myeloid malignancies, both 5-Aza-CdR and 5-Aza-CR have limited efficacy in treating solid tumors due to their plasma instability, cytotoxicity, and potentially mutagenic properties [85–87]. The instability of 5-Aza-CR and 5-Aza-CdR is attributed to hydrolysis and deamination, presenting a challenge for their clinical application. To address this issue, several cytidine analogues with improved stability and efficacy have been developed. Zebularine, which lacks an amino group in the 4-position of the pyrimidine ring, is less chemically labile and cytotoxic than the 5-Aza analogs. Studies have shown that it reactivates aberrantly silenced tumor suppressor genes in breast cancer cell lines [88] and inhibits polyp formation in female *MIN* mice [89]. Another method used to increase drug stability is to generate them as prodrugs. An example of this type of analogue is S110, a dinucleotide containing the 5-azacytosine ring that is less prone to deamination and less cytotoxic. S110 has been shown to induce *p1*6 expression by reducing DNA methylation in human xenografts [90].

In the past few years, tremendous efforts have been invested into broadening the application of 5-Aza-CdR and 5-Aza-CR to the treatment of solid tumors. A preclinical phase I trial in which 5-Aza-CR was subcutaneously administered to 19 dogs with naturally occurring invasive UC showed favorable tumor response. 72% of the dogs have demonstrated either partial remission or stable disease, meriting potential application of such treatment in humans [91].

## 8. Histone Deacetylase Inhibitors 

Another layer of epigenetic regulation includes posttranslational modifications of histones, which play an important role in gene expression by altering chromatin structure [92]. The type and location of histone modifications determine the conformation of chromatin. Certain modifications, such as H3K4me3 and H3K9 acetylation, are associated with euchromatin and make the DNA more accessible to the transcriptional machinery. Other modifications, such as H3K9me3 and H3K27me3, are associated with heterochromatin and make the DNA more condensed and less accessible to the transcriptional machinery [93, 94]. Cytosine methylation is associated with increased H3K9me3 and decreased H3 acetylation and H3K4me3 at gene promoters, leading to chromatin condensation and subsequent transcriptional silencing [95, 96]. The level of histone modifications is orchestrated by histone modifying enzymes, which add or remove specific histone marks to promote or hinder gene expression. A balance between these enzymes is necessary to maintain normal physiological conditions. Cancer cells lack this balance, as they typically overexpress histone deacetylases (HDAC), which results in a global reduction in histone acetylation [97].

More than 15 HDAC inhibitors are currently undergoing preclinical or clinical investigations for the treatment of both hematological malignancies and solid tumors, including UC [98]. Their common mechanism of action is the chelation of Zn^2+^ ion, which is critical to the enzymatic activity of HDAC [99]. To date, there are only 2 HDACIs that have been approved by the FDA for the treatment of cutaneous T-cell lymphoma, Vorinostat, also known as suberoylanilide hydroxamic acid (SAHA), and Romidepsin [94, 100]. HDACi have shown great clinical efficacy as single anticancer therapy only against certain hematological malignancies [101]. Although many have shown great potential for solid tumors in preclinical settings, in clinical settings they have generally yielded low responses [97, 102]. Among such HDACi, SAHA showed modest efficacy against UC in a phase I trial [103, 104]. To date, HDACi have demonstrated limited antitumor activity in UC and other solid tumors as a single agent; however, they have been well tolerated by patients [105]. *In vivo* studies have shown that a combinatorial treatment of HDACi and adenovirus-mediated gene therapy is more efficacious than either one alone, resulting in upregulation of the coxsackie and adenovirus receptor (CAR) gene, which is essential for the uptake of adenoviruses in target cells [106–108]. Such studies suggested the potential benefits of combining HDACi with other therapeutic agents to achieve a better therapeutic value in treating patients with UC. 

## 9. Combination Therapy

**Figure 2:**
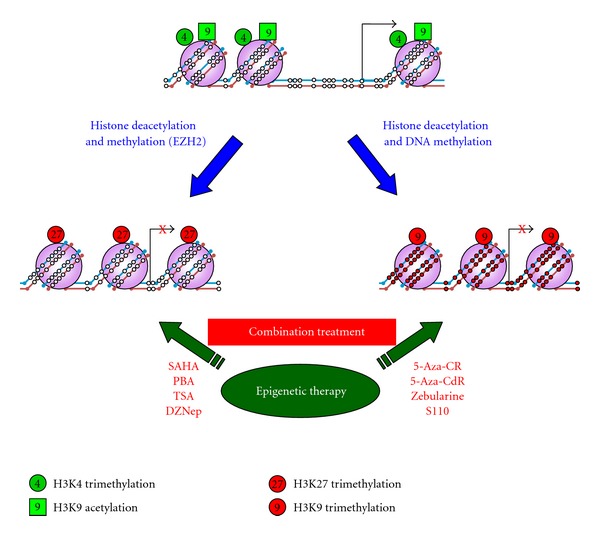
Epigenetic therapies can reverse aberrant epigenetic modifications in cancer. Genes that are expressed in normal cells, such as tumor suppressor genes, have an open chromatin structure, consisting of an unmethylated promoter, active histone marks, and a nucleosome-free region immediately upstream of the transcription start site. During tumorigenesis, genes can be silenced through one of the two silencing mechanisms: polycomb repressive complex (PRC) reprogramming and *de novo* DNA methylation. PRC-mediated silencing can be reversed upon treatment with EZH2 inhibitors, such as DZnep. The *de novo* methylation-mediated silencing can be reversed upon treatment with DNA methylation transferase inhibitors, such as 5-Aza-CdR, 5-Aza-CR, Zebularine, and S110. The therapeutic value of above reagents may be enhanced when combining with HDAC inhibitors, such as SAHA, PBA, and TSA. Open and closed circles represent unmethylated and methylated CpG sites, respectively.

The epigenome of UC is highly disrupted, featuring aberrant gene silencing either through the acquisition of DNA methylation or the repressive histone mark H3K27 trimethylation (Figure 2). The existence of these mechanisms suggests that the combination of DNMTi and HDACi may result in higher therapeutic efficacy. Both additive and synergistic effects have been reported with the combination of these two classes of epigenetic agents in patients with advanced hematological malignancies and solid tumors [32, 102]. However, the clinical utilization of combined epigenetic therapies is still in its early stages and more work is needed to elucidate the mechanism behind the increased clinical efficacy of sequential administration of DNMTi and HDACi in order to achieve an even greater synergistic effect.

The discovery of the vital role that aberrant epigenetic changes play in tumorigenesis as well as the reversibility of such changes has spurred great interest in the application of epigenetic therapies in cancer treatment with the primary goal of restoring aberrantly silenced genes. In addition, epigenetic therapies can also enhance the expression of cancer germline antigens, which are genes only expressed in germ cells and in a variety of cancers, including UC [109, 110]. Activating such genes increases the likelihood that tumor cells will be recognized and killed by antigen reactive CD8(+) T cells [111]. Epigenetic therapy can enhance the expression of cancer germline antigens, which are being actively pursued as vaccine targets. Therefore, combining epigenetic therapy with cancer germline antigen vaccine therapy may help amplify the therapeutic value of immunotherapy [110].

Despite its great promise, the application of epigenetic therapies to the treatment of UC and other types of solid tumors is still in its infant stage. Some of the issues that need to be resolved before this therapeutic approach is implemented includes the poor stability of the two FDA-approved DNMTi and the relapse of methylation after DNMTi treatment.

## 10. Conclusion and Future Directions

UC is as much a disease of disrupted epigenome as it is a disease of genetic mutations. Here, we have summarized the epigenetic abnormalities associated with UC, with an emphasis on DNA methylation. The presence of an epigenetic field defect, where DNA methylation of a significant number of genes is altered not only in primary tumors but also in the surrounding normal-appearing tissues, provides a plausible explanation for the high rate of UC recurrence. Since certain epigenetic alterations precede disease pathology, they have the potential to serve as excellent biomarkers for diagnosis, prognosis, and monitoring. Although a large number of highly specific markers, both genetic and epigenetic, have already been identified, they suffer from low sensitivity. The ability to detect methylation changes in readily obtainable urine samples opens the door for the development of sensitive and specific noninvasive methods for early detection and monitoring of UC. In addition to serving as biomarkers, epigenetic alterations are also excellent therapeutic targets. Epigenetic therapies, such as DNMTi and HDACi, aim at restoring the diseased epigenome to its normal state by reactivating aberrantly silenced genes. While they have shown promising results in both preclinical and clinical settings, their efficacy is still limited to a few hematological malignancies. Epigenetic therapies also reactivate cancer germline antigens, which can be recognized by the immune system, and, therefore, they could potentially enhance the therapeutic value of cancer germline antigen vaccines. Future work, including obtaining a greater understanding of the mechanisms of DNMTi and HDACi, is necessary to determine the extent of their utility in treating solid tumors. With the aid of readily available genome-wide DNA methylation and expression analyses and our rapidly accumulating knowledge regarding epigenetic regulation, the translation of these findings from the bench to the bedside in the near future is an obtainable goal.
